# Meta-analysis of predictive models to assess the clinical validity and utility for patient-centered medical decision making: application to the CAncer of the Prostate Risk Assessment (CAPRA)

**DOI:** 10.1186/s12911-018-0727-2

**Published:** 2019-01-07

**Authors:** Marine Lorent, Haïfa Maalmi, Philippe Tessier, Stéphane Supiot, Etienne Dantan, Yohann Foucher

**Affiliations:** 1 SPHERE (methodS in Patient-centered outcomes & HEalth ResEarch) U1246, INSERM, Nantes University, Tours University, Nantes, France; 2 Division of Clinical Epidemiology and Aging Research, Heidelberg University, Heidelberg, Germany; 3 Department of Radiotherapy, Institut de Cancérologie de l’Ouest René Gauducheau, Saint Herblain, France; 4 INSERM UMR892, Nantes University, Nantes, France; 5 Nantes University Hospital, Nantes, France; 6 IRS2, SPHERE U1246, 22 boulevard Bénoni Goullin, 44200 Nantes, France

**Keywords:** Meta-analysis, Prostate cancer, Patient-centered outcomes, Stratified medicine

## Abstract

**Background:**

The Cancer of the Prostate Risk Assessment (CAPRA) score was designed and validated several times to predict the biochemical recurrence-free survival after a radical prostatectomy. Our objectives were, first, to study the clinical validity of the CAPRA score, and, second, to assess its clinical utility for stratified medicine from an original patient-centered approach.

**Methods:**

We proposed a meta-analysis based on a literature search using MEDLINE. Observed and predicted biochemical-recurrence-free survivals were compared to assess the calibration of the CAPRA score. Discriminative capacities were evaluated by estimating the summary time-dependent ROC curve. The clinical utility of the CAPRA score was evaluated according to the following stratified decisions: active monitoring for low-risk patients, prostatectomy for intermediate-risk patients, or radio-hormonal therapy for high risk patients. For this purpose, we assessed CAPRA’s clinical utility in terms of its ability to maximize time-dependent utility functions (i.e. Quality-Adjusted Life-Years – QALYs).

**Results:**

We identified 683 manuscripts and finally retained 9 studies. We reported good discriminative capacities with an area under the SROCt curve at 0.73 [95%CI from 0.67 to 0.79], while graphical calibration seemed acceptable. Nevertheless, we also described that the CAPRA score was unable to discriminate between the three medical alternatives, i.e. it did not allow an increase in the number of life years in perfect health (QALYs) of patients with prostate cancer.

**Conclusions:**

We confirmed the prognostic capacities of the CAPRA score. In contrast, we were not able to demonstrate its clinical usefulness for stratified medicine from a patient-centered perspective. Our results also highlighted the confusion between clinical validity and utility. This distinction should be better considered in order to develop predictive tools useful in practice.

**Electronic supplementary material:**

The online version of this article (10.1186/s12911-018-0727-2) contains supplementary material, which is available to authorized users.

## Background

Prostate cancer (PC) is the most common cancer in men, accounting for approximately 10% of all male cancer deaths in economically developed countries [1]. In the long term, many patients experience biochemical recurrence (BCR), defined by an increase in prostate-specific antigen (PSA) levels [2], which can also lead to cancer-specific mortality [3]. Many developments have been made in predictive scoring systems [4–7] in order to avoid both over-treatment of patients at low-risk of cancer recurrence or death related to the cancer, and the under-treatment of patients at high-risk.

Among these scores, the Cancer of the Prostate Risk Assessment (CAPRA) score [8] was designed to predict the pre-operative risk of BCR-free survival after a radical prostatectomy (RP). RP represents the most widely performed curative therapy for patients with localized PC [9]. The CAPRA score is calculated from five variables measured before prostatectomy: the PSA level, the Gleason score, the clinical T stage, the percentage of positive prostate biopsies and the patient age at diagnosis. Meurs et al. [10] performed a substantial meta-analysis of seven studies and concluded that the CAPRA rule accurately predicts BCR-free survival at 3 years.

Nevertheless, despite its good predictive capacities, the clinical usefulness of the CAPRA score may be questioned. Firstly, the results of the meta-analysis of Meurs et al. [10] mainly consisted in risk ratios between expected and observed numbers of events among three CAPRA-based strata (0–2 for low risk, 3–5 for intermediate risk, and 6–10 for high risk). The aim was to evaluate the calibration compared to the initial study of Cooperberg et al. [8]. But the discriminative capacities were not evaluated. Secondly, the CAPRA thresholds used to define the three strata were arbitrarily defined, while the purpose of such a scoring system is to adapt the medical management of patients regarding the strata they belongs to. In two available online applications related to the CAPRA computation [11, 12], the following recommendations are proposed: routine surveillance for low risk patients, localized treatment (surgery or radiation alone, brachytherapy with or without external-beam therapy) for intermediate-risk patients, hormonal therapy or multimodal therapy (surgery with radiation, or radiation therapy with hormonal therapy) for high-risk patients. However, to the best of our knowledge, no study has assessed the clinical usefulness of the CAPRA score for stratified medical decision making.

We hypothesized that the clinical usefulness depends on its ability to bring benefits for patients by differentiating the following alternative therapies: the combined radio-hormonal therapy (RHT) for patients at high-risk of BCR, the RP for medium-risk patients and the active monitoring (AM) for low-risk patients. In this paper, from an update of the meta-analysis proposed by Meurs et al. [10], our objectives were i) to study the *clinical validity* of the CAPRA score, i.e. its prognostic capacities both in terms of discrimination and calibration, and ii) to evaluate its *clinical utility*, i.e. its usefulness for stratified medical decision making from an original patient-centered methodology that considers its corresponding benefits and drawbacks [13].

## Methods

### Search strategy

We conducted a literature search using the MEDLINE citation database to identify eligible studies by combining the following MeSH terms and keywords: “CAPRA score”, “biochemical recurrence” and “prostate cancer”. Since our analysis was an update of the meta-analysis of Meurs et al. [10] published in March 2012, we restricted our search after this date. No language restriction was performed. Cross-referencing was applied to complete the identification of other studies.

### Study selection

Two investigators (Y.F and E.D) independently screened the titles and abstracts of papers identified from the literature search. Irrelevant articles were excluded. The full text of each potentially relevant article was reviewed and checked independently by two persons (Y.F and E.D or H.M). Discrepancies between the three investigators were resolved by consensus. As illustrated in Table 1, a study was included in the analysis if it met the two following criteria: 1) prospective or retrospective study conducted in PC patients having experienced RP, with the preoperative CAPRA score as a predictor and BCR-free survival as the endpoint; 2) study delivering Hazard Ratios (HRs) and/or BCR-free survival curves stratified according to CAPRA values. When the same cohort was used in several publications, only the most recent study was selected if it contained the statistical indicators necessary for our meta-analysis.

**Table 1:** PICOS table related to the selection of the papers

Patient population	Patients with Prostate Cancer with available CAPRA score
Intervention	Having a radical prostatectomy
Comparison intervention	With or without control group
Outcomes	Biochemical-recurrence-free survival
Study type	Prospective or retrospective study presenting hazard ratio and/or survival curves

### Data extraction

From the eligible studies, two independent investigators (H.M and Y.F) collected the data independently. For each study, the following characteristics were collected: first author, year of publication, country, period of recruitment, duration of follow-up, study setting and design, sample size, age, reported outcomes, association between CAPRA and BCR-free survival (HRs and 95%CI), number of patients in each CAPRA-based strata, presence of survival curves and the corresponding number of at-risk patients, and the Harrell’s concordance index (C-index). Any disagreement was resolved by consensus. All statistical analyses were performed using the 3.1.1 version of the R software [14]. The survival probabilities were extracted from a digitalized picture by using the R packages *ReadImages* and *digitize* [15]. Many papers do not provide the number of at-risk patients over time, so we applied the method proposed by Parmar et al. [16] in order to obtain estimations.

### Assessing clinical validity

The association between the CAPRA score and BCR-free survival was evaluated by estimating pooled HRs which compared the high-risk category (CAPRA ≥6) and the intermediate-risk category (2 < CAPRA < 6) to the low-risk category of the CAPRA score (CAPRA≤2). When it was available, we used the category from 0 to 2 as the low-risk category, or otherwise we used the category 0 to 1. Collected HRs were pooled by applying a random-effects model and the DerSimonian-Laird estimator [17] (R package *metaphor*). Sensitivity analysis was conducted by eliminating a single study one-at-a-time. Publication bias was evaluated statistically by the Kendall’s tau and the Egger’s linear regression test of the intercept [18]. Heterogeneity between studies was assessed by Cochrane Q and I^2^ statistics [19] (R package *meta*). We also investigated potential heterogeneity by comparing pooled HRs stratified given possible heterogeneity factors: effective size, inclusion period and the geographic origin of the study. The outcome definition appeared homogeneous between studies, so there was no reason to test it as a heterogeneity factor.

Pooled survival curves per CAPRA-based strata were estimated by the distribution-free approach with random effects proposed by Combescure et al. [20]. The calibration was assessed graphically by comparing the pooled survival curves for each CAPRA strata to those estimated in the original paper by Cooperberg [8]. The discrimination capacities at 5-years post-RP were evaluated using the time-dependent summary ROC (SROCt) curves [21].

### Assessing clinical utility

We endorse a patient-centered approach to medical decision making by defining the clinical utility of a decision in terms of the expected Quality-Adjusted Life-Years (QALYs) it brings. QALYs combine into a single index, information about the length of life and health-related quality of life. They are computed by weighting each period of life (usually a year) by a utility score such that a score of zero indicates death and a score of one stands for perfect health [22]. The utility scores represent the individuals’ preferences over health states such that a higher score indicates a more preferred health state. Although QALYs were primarily designed for the conduct of economic evaluation, their composite nature may also be useful to inform clinical decision making [23].

More precisely, as demonstrated by Dantan et al. [24], the expected number of QALYs resulting from the use of a prognostic marker’s threshold to decide between two alternative therapies can be determined as follows. Let *T*, *X* and *κ* be the time-to-failure, the baseline prognostic marker under investigation to drive the treatment allocation, and a given threshold of *X*. Let *D*(*τ*) be the indicator of failure such that *D*(*τ*) = 1 if it occurs before the forecast horizon time *τ,* and *D*(*τ*) = 0 otherwise. Patient profiles may therefore be distinguished by combining their two possible initial strata according the marker *X* (*X* > *κ* or *X* ≤ *κ*) and the two possible outcomes (*D*(*τ*) = 1 or *D*(*τ*) = 0) over the time horizon under consideration. The optimal threshold *κ*^∗^ can be estimated as the threshold that maximizes the expected number of QALYs:

1 $$ {\sum}_{g\in \left\{X>\kappa, X\le \kappa \right\}}P(g)\left[{u}_{g,0}E\left(\min \left(T,\tau \right)|g\right)+{u}_{g,1}\left(\tau -E\left(\min \left(T,\tau \right)|g\right)\right)\right] $$

where *P*(*g*) is the proportion of patients in strata *g*, *E*(min(*T*, *τ*)| *g*) is the Restricted Mean Survival Time (RMST) up to time *τ* in the strata *g*, i.e. the average survival time when patients are followed up to *τ* [25] and (*u*_*g*, 0_, *u*_*g*, 1_) are the utility scores for health states *D*(*τ*) = 0 and *D*(*τ*) = 1 in the strata *g*. Therefore, calculating QALYs requires information about i) the survival probabilities and ii) the utility scores corresponding to the relevant health states.

#### Survival estimation

The RMST, *E*(min(*T*, *τ*)| *g*), which need to be estimated for all possible thresholds *κ* of the marker *X*, was estimated as the area under the corresponding survival curve *S*(*t*| *g*) until time *τ*. We used the methodology previously proposed by Combescure et al. [21]. Survival probability at time *t* given strata *g* is defined as follows and numerically calculated using the 30-points Gauss–Legendre quadrature:

2 $$ S\left(t|g=\left\{X>\kappa \right\}\right)=\frac{\int_{\kappa}^{\infty}\exp \left(-{\int}_0^t{E}_{\nu}\left[\lambda \left(u|x,\nu \right)\right] du\right)E\left[f\left(x|\omega \right)\right] dx}{\int_{\kappa}^{\infty }E\left[f\left(x|\omega \right)\right] dx} $$

In eq. 2, the distribution of the marker *X*, *f*(*x*| *ω*_*i*_), is estimated according to a log-normal distribution interval-truncated in-between 0 and 10 (i.e. the CAPRA range), *ω*_*i*_~Ν(0, *σ*_*ω*_) is a random effect of the mean for the study *i* and *σ*_*ω*_ its standard error. The instantaneous risk function of BCR, namely *λ*(*t*| *x*, *ω*_*i*_), is estimated according to a flexible piecewise constant risk function:

3 $$ \lambda \left(t|x,{\nu}_i\right)=\mathit{\exp}\left[{\nu}_i+{\sum}_{l=0}^L\left({\beta}_{l,1}+{\beta}_{l,2}\right)\times 1\left\{t>{\tau}_l\right\}\right] $$

where (*β*_*l*, 1_, *β*_*l*, 2_) are the regression coefficients specific to the interval *l* ∈ {1, …, *L*} (*L* being the number of time intervals), *υ*_*i*_~Ν(0, *σ*_*υ*_) is a random effect of the mean for the study *i* and *σ*_*υ*_ its standard error. To obtain *f*(*x*| *ω*_*i*_) and *λ*(*t*| *x*, *ν*_*i*_), we used the R package *nlme* based on the maximization of the restricted log-likelihood.

The survival probabilities were estimated from articles including patients with RP. For a stratified decision, we considered alternative therapies (AM and RHT) that will impact the survival probabilities. We studied several plausible scenarios in terms of decreasing length of life: a RMST loss of 10% at 5 years for AM versus RP [26] and in-between 20 to 40% for RP versus RHT [27–29].

#### Utility scores determination

Following Koerber et al. [30], we assumed that the baseline utility for PC patients can be approximated by an age-adjusted utility score of the general population. This baseline utility is then combined with the utilities of various health conditions (symptoms of prostate cancer and treatments) using a multiplicative model. Although there is no consensus about the appropriate method that should be used, there is recent evidence in favor of the multiplicative model for combining scores for health states compared to the alternative model [31, 32].

The event predicted by the CAPRA score is the first failure between disease progression (biochemical recurrence or distant metastasis) and death. For each treatment group (RP, AM, RHT), we calculated the expected utility before this event as the product of age-adjusted baseline utility and the utilities of symptoms associated with the treatment group weighted by their repartition. We calculated the corresponding utility after the event as the product of utility before the event and the utilities of disease progression or death weighted by their repartition. Supplementary information on the precise method of utility calculation is provided in the Additional file 1.

Numerous studies have published utility scores for health states related to PC assessed using various methods [33–38]. We chose the utility scores estimated by Stewart et al. [37] on a sample of patients aged 60 (the mean/median patient age in the included study in our meta-analysis centered around this value, Table 3) and over. These scores were deemed the most suitable for our study because they were directly assessed from patients and they were reported for both single symptoms and for comorbid health conditions. The raw utility scores and their sources are reported in Table 2. Since there was no utility value for active monitoring in the study proposed by Stewart et al. [37], we arbitrarily chose a score of 0.98 which seems in line with recent evidence suggesting that men undergoing active surveillance have high utility scores [33]. Repartition of each symptom and subtype of event given the treatment group are based on a literature search and are detailed in the Additional file 1.

**Table 2:** Sources and estimation of raw utility scores^a^

	Value	Source
Baseline utility value for age 60	0.84	Ara and Brazier [32]
Sexual dysfunction	0.89	Stewart et al. [37]
Sexual dysfunction and urinary incontinence	0.78	
Impotence and bowel dysfunction	0.57	
Impotence, urinary incontinence, and bowel dysfunction	0.45	
Local disease progression	0.67	
Metastatic cancer	0.25	
Active monitoring	0.98	Authors’ assumption

^a^The table reports ‘raw’ scores. The final scores used in our calculations were obtained by combining the baseline score with these raw scores using a multiplicative model. For instance, the final score for sexual dysfunction is obtained by multiplying the score in Stewart et al. by our baseline utility value (see the Additional file 1)

Finally, for patients alive without BCR, the utility scores were estimated at 0.79 under AM, 0.76 under RP and 0.72 under RHT. By merging the utilities related to the combined event (disease progression or death), the expected utility scores after the treatment failure were assessed at 0.34 under AM, 0.24 under RP and 0.20 under RHT. A sensitivity analysis was carried out by varying the patient age between 55 and 75 years. The conclusions of the analyses were unchanged (data not shown)

## Results

### Description of the selected studies

**Fig. 1:**
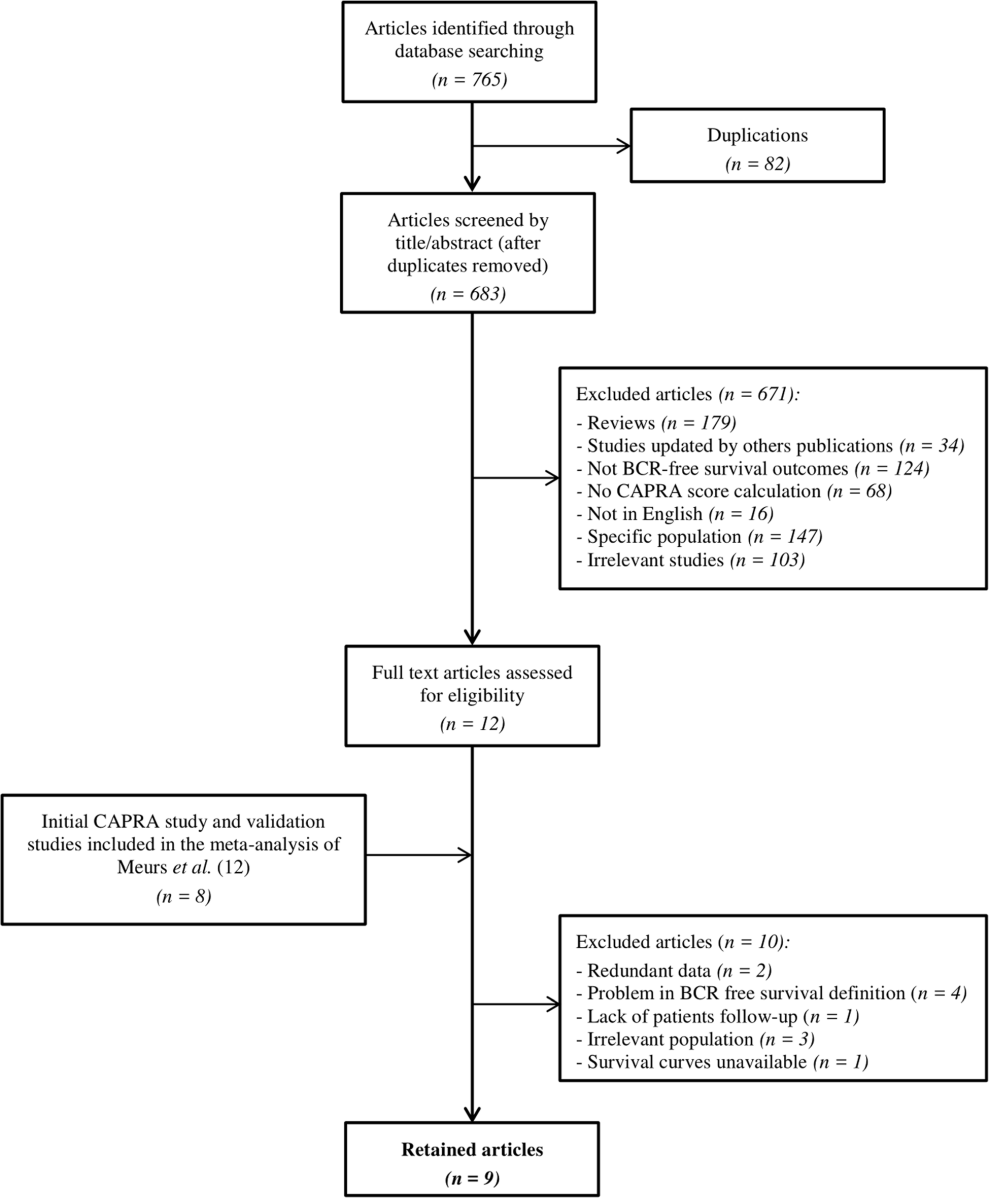
The flowchart describing the 9 selected articles of the meta-analysis

A flowchart of the selected studies is presented in Fig. 1. We identified 683 manuscripts of which only 12 underwent full text review. No additional study was identified by cross-referencing. We also considered the 8 validation studies included in the meta-analysis of Meurs et al. [10] Among these 20 articles, we finally retained 9 studies: the original study of Cooperberg et al. [8], 5 studies [39–43] published between 2005 and 2012 and selected by Meurs et al. [10], and 3 additional studies [44–46] published more recently. The study proposed by Tamblyn et al. [47] in 2011, selected by Meurs et al. [10], was excluded because the authors did not present BCR-free survival curves according to CAPRA values. The work of Lughezzani et al. [48] in 2010, selected by Meurs et al. [10], was excluded because the same population with a longer follow-up was used by Budaus et al. [44] in 2012. Furthermore, the latter included all the statistical indicators necessary for our meta-analysis.

The main features of the 9 eligible studies (15,908 patients) are summarized in Table 3. All studies were based on prospective cohorts of patients treated by RP. The sample size per study ranged from 115 to 6737 patients. The mean age ranged from 58.0 to 66.4 years. Four studies were conducted in the USA, 2 in Germany, 2 in Japan, 1 in Korea and 1 in Australia.

**Table 3:** Description of the 9 studies included in the meta-analysis

Study	Country, Period, Follow-up	Effective size, Ethnicity, Age	Definition of BCR	CAPRA (N)	HR (95%CI)	C-index
Cooperberg et al., 2005 [8]	USA, 1992–2001, median = 24 m	1439, 88% Caucasian 8.4% African-American mean = 62y (no SD)	2 consecutive PSA ≥ 0.2 ng/mL or secondary treatment for elevated postoperative PSA	0–1 (401) 2 (432) 3 (296) 4 (155) 5 (84) 6 (43) 7–10 (28)	1.28 (0.79–2.08) 2.36 (1.49–3.72) 2.38 (1.40–4.03) 3.32 (1.89–5.80) 7.11 (3.84–13.15) 17.38 (9.92–30.46)	0.660
Cooperberg et al., 2006 [39]	USA, 1988–2004, mean = 42 m	1309, 59% Caucasian, mean = 61.9y (±6.6)	1 PSA > 0.2 ng/mL or 2 consecutive PSA ≥ 0.2 ng/mL or secondary treatment for elevated postoperative PSA	0–1 (324) 2 (329) 3 (291) 4 (158) 5 (108) 6 (76) 7–10 (60)	1.89 (1.25–2.85) 2.75 (1.85–4.10) 3.29 (2.15–5.04) 4.51 (2.89–7.05) 7.19 (4.58–11.30) 9.90 (6.34–15.46)	0.680
May et al., 2007 [42]	Germany, 1992–2005, mean = 56 m	1296, 100% Caucasian, mean = 63.7y (±5.5)	1 PSA > 0.2 ng/mL, 2 PSA ≥ 0.2 ng/mL or secondary treatment for elevated postoperative PSA	0–1 (130) 2 (297) 3 (265) 4 (222) 5 (140) 6 (92) 7–10 (150)	unknown	0.810
Zhao et al., 2008 [43]	USA, 1984–2006, median = 4y	6737, 91% Caucasian, mean = 58y (no SD)	1 PSA level > 0.2 ng/mL	0–1 (2796) 2 (1937) 3 (1005) 4 (463) 5 (306) 6 (161) 7–10 (69)	2.24 (1.83–2.74) 3.69 (3.01–4.54) 8.61 (6.96–10.65) 9.52 (7.57–11.97) 13.41 (10.40–17.29) 18.96 (13.87–25.92)	0.760
Loeb et al., 2012 [41]	USA, 2003–2009, median = 34 m	726, 93.5% Caucasian, mean = 59.3y (no SD)	Repeated PSA ≥ 0.2 ng/mL, secondary treatment for elevated postoperative PSA	0–1 (441) 2 (263) 3 (113) 4 (80) 5 (57) 6 (23) 7–10 (13)	1.1 (0.4–3.0) 4.1 (1.7–10.0) 5.1 (2.1–12.5) 10.5 (4.4–24.7) 12.2 (3.8–38.9) 47.8 (17.1–133.2)	0.764
Ishizaki et al., 2011 [40]	Japan, 1999–2010, mean = 38 m	211, 100% Japanese, mean = 62.2y (±5.8)	2 PSA ≥ 0.2 ng/mL, secondary treatment for elevated postoperative PSA	0–2 (85) 3–5 (106) 6–10 (20)	2.14 (1.19–3.86) 9.14 (4.30–19.44)	0.755
Budaus et al., 2012 [44]	Germany, 1992–2009, mean = 56 m	2937, unknown, median = 64y (48–74)	1 PSA level ≥ 0.2 ng/mL	0–2 (1280) 3–5 (1270) 6–10 (387)	3.1 (2.4–3.9) 7.0 (5.5–9.0)	0.714
Yoshida et al., 2012 [46]	Japan, 1995–2008, median = 44 m	503, 100% Japanese, median = 65y (47–76)	1 PSA ≥ 0.2 ng/mL followed by a 2nd PSA higher, radiotherapy or hormonal therapy for the postoperative PSA elevation	0–2 (138) 3–5 (257) 6–10 (108)	1.67 (0.93–2.99) 3.97 (2.18–7.24)	0.673
Seo et al., 2014 [45]	Korea, 2008–2013, median = 13 m	115, 100% Korean, mean = 66.4y (±6.5)	2 PSA ≥ 0.2 ng/mL, additional treatment more than 6 months after RP	unknown	unknown	0.770

*BCR* Biochemical Recurrence, *CAPRA* Cancer Prostate Risk Assessment, *95%CI* Confidence Interval, *C-index* Harrell’s concordance index, *HR* Hazard Ratio, *m* months, *SD or ±* Standard Deviation, *PSA* Prostate-Specific Antigen, *RP* Radical Prostatectomy, *y* years

### Association between the CAPRA score and BCR-free survival

**Fig. 2:**
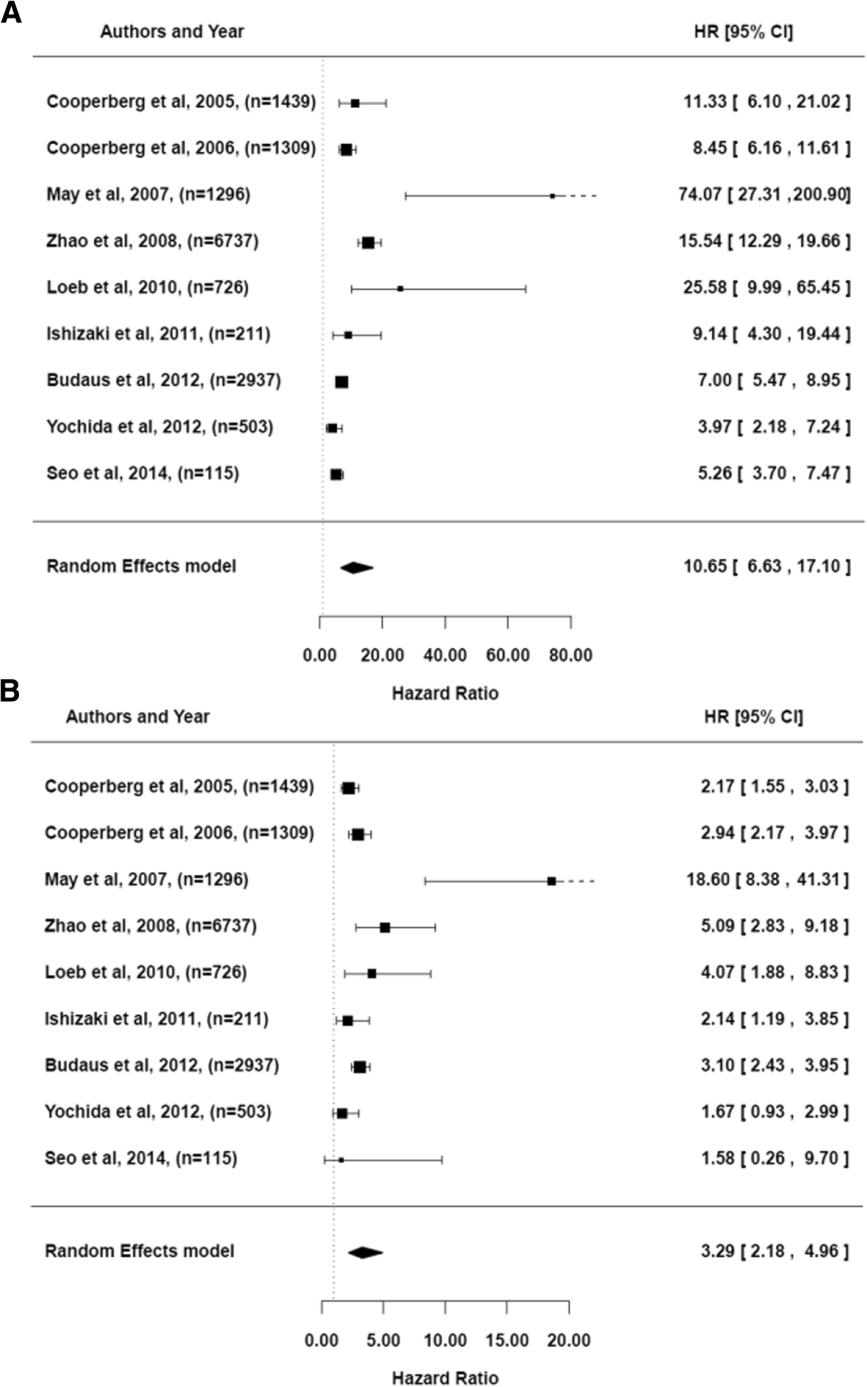
The forest plots for pooled hazard ratios. **a**) High-risk group (CAPRA ≥6) versus the low-risk group (CAPRA ≤2). **b**) Intermediate-risk group (2 < CAPRA < 6) versus the low-risk group (CAPRA ≤2)

The 9 retained articles [8, 39–46] provided HRs or survival curves (from which one can re-estimate the HRs) according to CAPRA-based strata. As illustrated in Fig. 2, the pooled HR of patients at high-risk (CAPRA≥6) versus those at low-risk (CAPRA≤2) was 10.65 [95%CI from 6.63 to 17.10]. The HR was 3.29 [95%CI from 2.18 to 4.96] for patients at intermediate-risk compared to those at low-risk. The analysis indicated a non-significant publication bias (Kendall’s test statistic = 0.28, *p* = 0.3585 for the two group comparisons; Egger’s tests = 1.06, *p* = 0.210 for high versus low risk groups; Egger’s tests = 0.46, *p* = 0.6474 for intermediate versus low risk groups) and a large heterogeneity among the studies (Cochrane Q = 63.16, *p* < 0.0001 for high versus low risk groups; Cochrane Q = 33.08, p < 0.0001 for intermediate versus low risk groups; I^2^ = 91.18 and 84.25% for the 2 respective group comparisons). As illustrated in Table 4, one cannot identify any significant reason to explain this heterogeneity.

**Table 4:** Investigation of the heterogeneity of the HRs according to CAPRA-based strata

	Number of studies per subgroup	Pooled HR (High versus Low) per subgroup	*p*-value	Pooled HR (Intermediate versus Low) per subgroup	*p*-value
Effective size	> 1000: 5 ≤ 1000: 4	14.60 [7.24–29.43] 8.13 [4.13–16.01]	0.240	4.45 [2.32–8.54] 2.35 [1.59–3.47]	0.098
Inclusion period	Inclusion start ≥1999: 3 Inclusion start < 1999: 6	10.37 [5.00–21.49] 11.78 [5.83–23.80]	0.804	2.78 [1.83–4.24] 3.78 [2.04–7.00]	0.419
Geographic origin	American: 4 Other: 5	13.37 [9.25–19.32] 9.75 [4.02–23.60]	0.518	3.34 [2.41–4.61] 3.39 [1.48–7.77]	0.971

### Clinical validity of the CAPRA score up to five years post-prostatectomy

The area under the SROCt curve (AUC) at 5 years post-prostatectomy was 0.73 [95%CI from 0.67 to 0.79]. The AUCs for each study ranged from 0.67 [95%CI from 0.61 to 0.73] to 0.76 [95%CI from 0.71 to 0.81], indicating acceptable discriminative capacities of the CAPRA score.

**Fig. 3:**
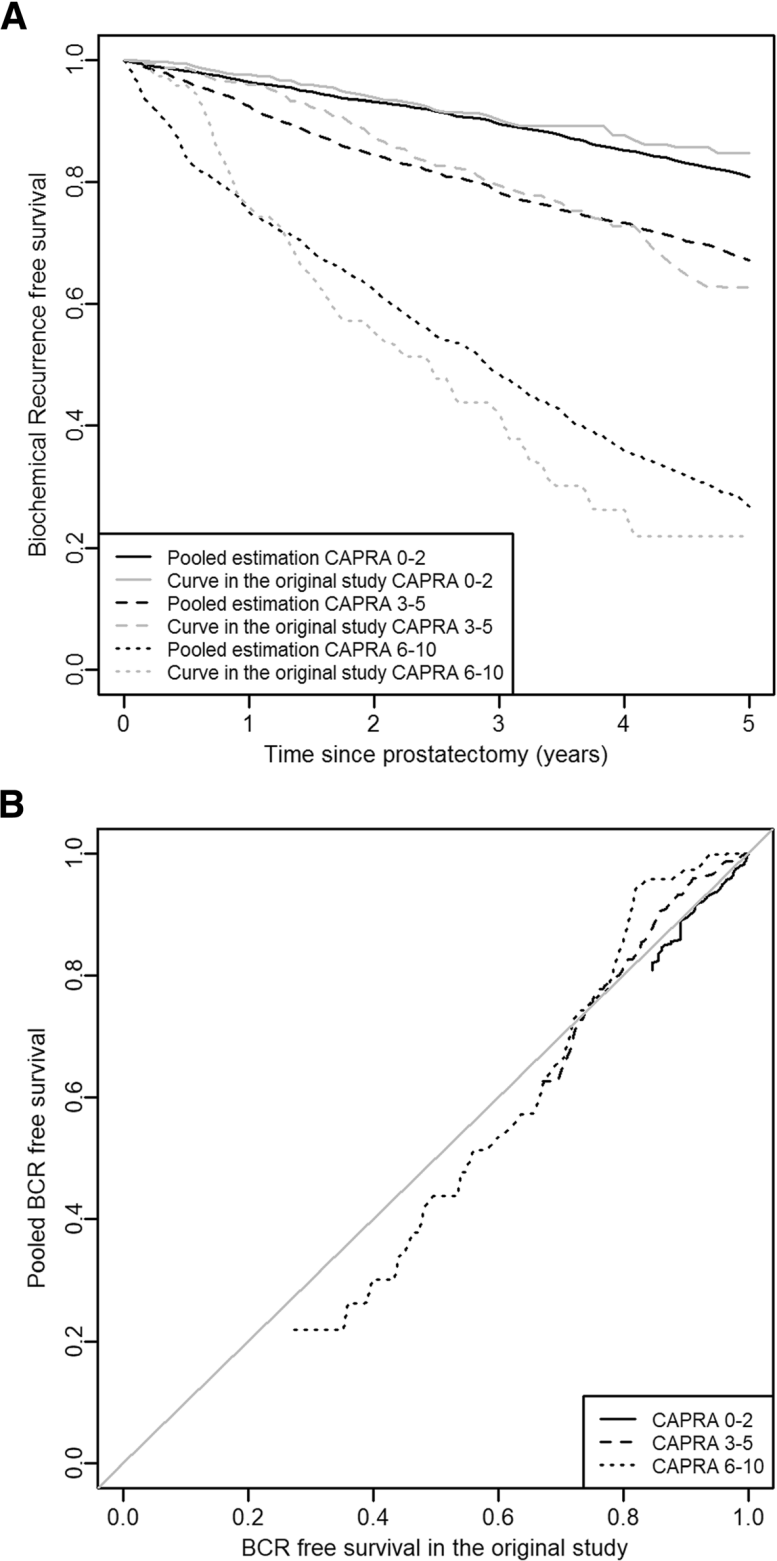
Calibration plots. **a** The pooled survival curves according to CAPRA-based strata compared to the survival curves from the original study. **b** Pooled survival versus observed survival in the original study

Seven validation studies [39–41, 44–46] with survival curves according to the 3 CAPRA-based strata were considered. As illustrated in Fig. 3, patients at low-risk have 80.8% chance [95%CI from 70.8 to 86.6] of not suffering a BCR or a death within the 5 years post-RP. In contrast, patients at high-risk have a 73.2% risk [95%CI from 61.7 to 86.6] of suffering a BCR or death within 5 years post-RP. Note that in the original study, the 5-year BCR-free survival probabilities in low-, intermediate-, and high-risk strata were 84.7, 62.7 and 21.8%, respectively (Fig. 3, 95%CI were not provided). In comparison to the pooled survival curves, the calibration of the CAPRA score appeared acceptable.

### Clinical utility of the CAPRA score for deciding AM instead of RP

Only patients with a CAPRA score strictly lower than 6 were included since AM is not proposed to high-risk patients (i.e. CAPRA≥6). The maximum number of QALYs was obtained when RP is proposed to all patients. In other words, for all the CAPRA thresholds in-between 1 and 5, the expected gain in terms of QoL related to AM was insufficient to balance the associated decrease in the BCR-free survival. More precisely, the mean number of QALYs, i.e. the expected equivalent of years lived in perfect health, was 42.4 months if all patients are treated by RP. In contrast, when patients with a CAPRA score lower than 2 (the usual threshold separating low and intermediate-risk strata) are treated by AM and the other ones by RP, the mean number of QALYs was 41.7 months. Therefore, for a cohort of 100 patients, this stratification was associated with an expected loss of 70 months in perfect health compared to treating all patients by RP.

### Clinical utility of the CAPRA score for deciding RHT instead of RP

Only patients with a CAPRA score strictly higher than 2 were included since treatment by RHT is not proposed to low-risk patients (i.e. CAPRA≤2). The maximum number of QALYs was obtained by proposing RHT to all patients: 41.6 months in perfect health state. Regardless of the CAPRA threshold, all the stratified medical decision making resulted in lower numbers of QALYs. For instance, by using the usual CAPRA-based stratification (CAPRA< 6 for intermediate risk strata versus CAPRA≥6 for high risk strata), we estimated that the mean number of life-years in perfect health was 39.6 months. Therefore, for a cohort of 100 patients, the use of CAPRA threshold at 6 for stratifying the medical decision between RP and RHT would have been associated with a loss of 200 months in perfect health state compared to treating all patients by RHT.

## Discussion

Prognostic tools are essential in PC to adapt the medical management regarding the risk-level of patients [49–51]. In this context, our meta-analysis aimed to precisely describe the usefulness of the CAPRA score for stratified medicine.

Our results were obtained by analyzing 9 studies. In agreement with the literature and by considering the three usual CAPRA-based strata, we validated the association between the CAPRA score and the BCR-free survival (HR intermediate versus low = 3.29 [95%CI from 2.18 to 4.96] and HR high versus low = 10.65 [95%CI from 6.63 to 17.10]). We additionally described the CAPRA score as a marker with a robust capacity to discriminate BCR or dead patients at 5-year post-RP from alive patients without BCR at this time (AUC = 0.73 [95%CI from 0.67 to 0.79]). In terms of calibration, we also reported acceptable properties up to 5 years post-RP. However, despite this validity, we were unable to demonstrate the clinical utility of the CAPRA score in regards to the following stratified medical decision making: AM for low-risk patients, RP for intermediate-risk patients and RHT for high-risk patients.

Our study highlights the important confusion between clinical validity and utility. Indeed, even if the relevance of stratified medical decision making partially depends on the prognostic capacities of a biomarker or a scoring system, other dimensions are also important to consider, in particular the consequences of both quantity and quality of life, and on trade-offs between them. Several recent studies discussed the importance of considering these two dimensions [23, 52, 53]. We also believe that better understanding of these dimensions in studies related to predictive biomarkers or scoring systems for stratified medicine can improve the transfer of such tools into clinical practice.

From a methodological point of view, the large majority of papers in stratified medicine have focused on the evaluation of prognostic capacities, mainly by estimating area under ROC curves. In contrast, our results illustrate the necessity for a paradigm shift towards the evaluation of clinical usefulness, by estimating time-dependent utility function for instance [24].

Alternative decision-making approaches have been proposed that incorporate the consequences of clinical choices when assessing the usefulness of diagnostic tests or of prediction models. For instance, the widely used Decision Curve Analysis (DCA) computes the net benefit of a medical decision as the difference between its benefits and harms [54]. In DCA, the benefits and harms are obtained by asking the physicians for the probability threshold, i.e. the rate at which the patients are willing to trade-off between the harms and the benefits of a medical decision. A decision curve then plots the net benefit of a decision against the net benefits of “treating all” or “treating none” for the various possible values of the probability threshold. Although DCA may be easier to implement compared to our QALY-based approach, the two approaches may not lead to similar conclusions as they differ importantly. DCA relies on the physicians’ prediction about the value of outcome [55], whereas patients’ utility scores come from those experiencing (of having experienced) the health conditions under consideration. There is no guarantee that the physicians’ judgments accurately reflect the views of the patients. Several studies found differences between the preferences of the patients and physicians for various conditions [56], especially in prostate cancer [57]. Although our QALY-based approach may be more in line with patients’ preferences than DCA, the assessment of utility scores raises practical difficulties and published utility values are quite heterogeneous. Nevertheless, we believe that addressing these difficulties in future research may offer a more promising way to develop patient-centered approaches of medical decision making rather than relying solely on physicians’ judgements.

It appears important to underline that the concept of clinical utility is multidimensional. It cannot be summarized into a single measure, whether it is QALYs or DCA. As explained by Smart [13], one can judge the concept of utility by using four components: appropriate, accessible, practicable, and acceptable. QALYs or DCA mainly concern the first one.

Our study nevertheless has some limitations. First, we identified a significant heterogeneity between the studies retained in our meta-analysis. We explored the possible causes of this heterogeneity, but we were not able to identify any significant reason. Second, the estimations of the SROCt curve [21] and the time-dependent utility function [24] both depend on many parametric assumptions. As with any meta-analysis on aggregated data, the corresponding limitation being listed by Lyman and Kuderer [58], it would have been preferable to collect the individual data necessary for such an analysis. To limit this issue, we have also non-parametrically adapted the two estimators after having reconstructed individual data (data not shown). The corresponding results were similar to the ones described in the main text. Third, numerous hypotheses are required to define the potential consequences of each alternative treatment (AM, RP and RHT) in terms of both quantity and quality of life. We attempted to be as realistic as possible in defining these hypotheses on data recently published in this field. Fourth, we did not study RP (for medium-risk patients) and RP + RHT (for high-risk patients). The reason is that it would require information about the removed tumor that is not included in the CAPRA score. Cooperberg et al. proposed the CAPRA-S score [59], as an extension of the CAPRA score that consider information about the tumor (surgical margin, seminal vesicle invasion, extracapsular extension, lymph node invasion). However, an analysis based on the CAPRA-S would be beyond the scope of our paper. Fifth, we separately investigated the discriminative capacities and the calibration of the CAPRA score. It would have been relevant to also estimate the global prognostic performance of the score, for instance by estimating the Index of Prognostic Accuracy, as recently proposed by Kattan and Gerds [60]. Nevertheless, the corresponding pooled estimation from a published study does not exist to our knowledge and constitutes interesting perspective for future methodological developments.

## Conclusions

Our study validated the prognostic capacities of the CAPRA score. Nevertheless, based on a QALYs maximization approach, our results challenged its clinical usefulness to beneficially stratify patients between three common therapeutic strategies: RHT for high-risk, RP for intermediate-risk, and AM for low-risk patients. Our results highlight the confusion between prognostic capacities and clinical usefulness. It calls for a better consideration of this distinction and for a paradigm shift from clinical validity to clinical utility in studies related to stratified medicine.

## Additional file

Additional file 1: Calculation of the utility scores for the different patient health and care states. The corresponding text explains the calculation of the utility scores for the different patient health and care states. (PDF 109 kb)

## Abbreviations

AM: Active monitoring
AUC: Area under the SROCt Curve
BCR: BioChemical Recurrence
CAPRA: CAncer of the Prostate Risk Assessment
HR: Hazard ratio
PSA: Prostate-specific antigen
QALY: Quality-Adjusted Life-Year
RHT: Radio-Hormonal Therapy
RP: Radical Prostatectomy
SROCt: Time-dependent Summary ROC
