# Supplementary material for: Meta-analysis of predictive models to assess the clinical validity and utility for patient-centered medical decision making: application to the CAncer of the Prostate Risk Assessment (CAPRA)
Source: BMC Med Inform Decis Mak. 2019 Jan 7;19:2. doi: 10.1186/s12911-018-0727-2 (PMC6323757; doi:10.1186/s12911-018-0727-2)
Supplement: Supplementary file 1 — Calculation of the utility scores for the different patient health and care states. The corresponding text explains the calculation of the utility scores for the different patient health and care states. (PDF 109 kb) [file 12911_2018_727_MOESM1_ESM.pdf]

Online supplementary information: calculation of the utility scores for the different patient health and care states

The event predicted by the CAPRA score is the first failure between disease progression (biochemical recurrence or distant metastasis) and patient death. In order to use the same terminology regardless of the treatment options (Radical Prostatectomy RP, Active Monitoring AM, and combined radio-hormonal therapy RHT), we divided the disease progression (DP) into two separate events: local disease progression (LDP) and distant metastasis (MET). Let U(base) be the utility for a 60 year-old man with PC. In Ara et al. [1], U(base) was estimated at 0.837 for the general UK population. To estimate utilities of health states related to PC, we combined the raw utility scores of Table 2 with U(base) by Koerber *et al.* [2].

Utility for a 60 year-old man with PC treated by RP prior to experiencing an event.

The most frequent side effects observed in patients treated by RP are Sexual Dysfunction (SD) and Urinary Incontinence (UI) [3]. We note U(SD) and U(SD,UI) the utilities for patients with SD alone, and with SD and UI, respectively. According to Stewart *et al.* [4], who assessed 162 PC patients, U(SD) equals 0.89 and U(SD,UI) equals 0.78. Let P(SD|RP) and P(SD,UI|RP) be the proportions of patients suffering respectively from SD alone and from SD and UI, among patients treated by RP. In the report of the UK National Screening Committee [3], P(SD|RP) has been estimated at 0.58 and P(SD,UI|RP) at 0.11. Let U(RP,before) be the utility for a 60 year-old man with prostate cancer, who has been treated by RP, before he experienced an event (disease progression or death). Following the multiplicative model one obtains:

$$
\begin{aligned}
U(RP,before) &= U(base) * \{ P(SD|RP)*U(SD) + P(SD,UI|RP)*U(SD,UI) \\
&\quad + (1 - P(SD|RP) - P(SD,UI|RP)) \} \\
&= 0.837 * \{0.58 * 0.89 + 0.11 * 0.78 + (1 - 0.58 - 0.11)\} \\
&= 0.76
\end{aligned}
$$

Utility for a 60 year-old man with PC treated by RP after he experienced an event

Let P(D|RP) be the proportion of deaths among the two possible events (D for death and DP for disease progression) in patients with RP. Bill-Axelson *et al.* [5] estimated this conditional probability at 0.31. Among patients who have disease progression, 50% should develop metastasis (MET): P(MET|DP,RP) = 0.50. The other half having suffered a local disease progression (LDP). According to Stewart *et al.* [4], the respective utilities are: U(MET) = 0.25 and U(LDP) = 0.67. Let U(RP,after) be the utility for a 60 year-old man with PC, who has been treated by RP and subsequently experienced an event:

$$U(RP,after) = U(RP,before) * \{ P(D|RP)*0 + (1 - P(D|RP))$$

$$* \ (P(MET|DP,RP)*U(MET) + (1 - P(MET|DP,RP))*U(LDP)) \}$$

$$= 0.76 * \{0.31 * 0 + (1-0.31) * (0.50 * 0.25 + 0.50 * 0.67)\}$$

$$= 0.24$$

Utility for a 60 year-old man with PC treated by AM before he experienced an event

Let P(SD|AM) be the proportion of patients who suffer from SD among patients treated by AM. According to the report of the UK National Screening Committee [3], P(SD|AM) can be assumed at 0.35. Let X be the unknown stress factor of patients who are actively monitored, living with the disease without active treatment. We arbitrarily set X equals 0.98. If U(AM,before) represents the utility for a 60 year-old patient with PC, who has been followed by AM, and before he experienced an event; we have:

$$U(AM,before) = U(base) * \{ P(SD|AM)*U(SD) + (1-P(SD|AM) \} * X$$

$$= 0.837 * \{0.35 * 0.89 + (1 - 0.35)\} * 0.98$$

$$= 0.79$$

Utility for a 60 year-old men with PC treated by AM after he experienced an event

Let P(D|AM) be the proportion of deaths among the events occurring in patients followed by AM. According to the study of Bill-Axelson *et al.* [5], one can assume that P(D|AM) = 0.22, and that among patients who have disease progression, 27% have developed metastasis: P(MET|DP,AM) = 0.27. Let U(AM,after) be the utility for a 60 year-old man with PC, who has been treated by AM after he experienced an event:

$$U(AM,after) = U(AM,before) * \{ P(D|AM)*0 + (1- P(D|AM))$$

$$* \ (P(MET|DP,AM)*U(MET) + (1 - P(MET|DP,AM))*U(LDP)) \}$$

$$= 0.79 * (0.22 * 0 + 0.78 * (0.27 * 0.25 + 0.73 * 0.67))$$

$$= 0.34$$

Utility for a 60 year-old men with PC treated by RHT before he experienced an event

Three types of serious adverse events can be considered after RHT: SD, UI and Bowel Complications (BC). According to the report of the UK National Screening Committee [3], among patients treated by RHT, 84% suffer from SD alone (P(SD|RHT) = 0.84), 7% suffer from SD and BC (P(SD,BC|RHT) = 0.07), and 3% suffer from SD, BC and UI (P(SD,BC,UI|RHT) = 0.03). According to Stewart *et al.* [4], U(SD,BC) = 0.57 and U(SD,BC,UI) = 0.45. If U(RHT,before) represents the utility for a 60 year-old patient with PC, who has been treated by RHT, and before he experiences an event; we have:

$$U(RHT,before) = U(base) * \{ P(SD|RHT)*U(SD) + P(SD,BC|RHT) * U(SD,BC)$$

+ P(SD,UI,BC|RHT) * U(SD,BC,UI)

+ (1 – P(SD|RHT)- P(SD,BC|RHT) - P(SD,UI,BC|RHT)) }

= 0.837 * (0.84*0.89 + 0.07*0.57 + 0.03*0.45 + (1 - 0.84 - 0.07- 0.03))

= 0.72

Utility for a 60 year-old men with PC treated by RHT after he experiences an event

Let P(D|RHT) be the proportion of deaths among the events occurring in patients treated by RHT. According to the study of Bolla *et al.* [6], we assumed that P(D|RHT) equals 0.40, and that among patients who have disease progression, 50% have developed metastasis (P(MET|DP,RHT) = 0.50). The other half had a local disease progression. If U(RHT,after) represents the utility for a 60 year-old patient with PC, who has been treated by RHT, and after he had experienced an event; we have:

U(RHT,after) = U(RHT,before) * { P(D|RHT)*0 + (1- P(D|RHT))

* (P(MET|DP,RHT) * U(MET) + (1- P(MET|DP,RHT)) * U(LDP)) }

= 0.72 * (0.40 * 0 + 0.60 * (0.50 * 0.25 + 0.50 * 0.67))

= 0.20

References

[1] Ara R, Brazier J. Estimating Health State Utility Values for Comorbidities. PharmacoEconomics 2017;35:89–94. doi:10.1007/s40273-017-0551-z.

[2] Koerber F, Waidelich R, Stollenwerk B, Rogowski W. The cost-utility of open prostatectomy compared with active surveillance in early localised prostate cancer. BMC Health Serv Res 2014;14:163. doi:10.1186/1472-6963-14-163.

[3] Hummel S, Chilcott J. Report to the UK National Screening Committee. University of Sheffield School of Health and Related Research (ScHARR); 2013.

[4] Stewart ST, Lenert L, Bhatnagar V, Kaplan RM. Utilities for prostate cancer health states in men aged 60 and older. Med Care 2005;43:347–55.

[5] Bill-Axelson A, Holmberg L, Ruutu M, Garmo H, Stark JR, Busch C, et al. Radical prostatectomy versus watchful waiting in early prostate cancer. N Engl J Med 2011;364:1708–17. doi:10.1056/NEJMoa1011967.

[6] Bolla M, Van Tienhoven G, Warde P, Dubois JB, Mirimanoff R-O, Storme G, et al. External irradiation with or without long-term androgen suppression for prostate cancer with high metastatic risk: 10-year results of an EORTC randomised study. Lancet Oncol 2010;11:1066–73. doi:10.1016/S1470-2045(10)70223-0.
